# Association of post-thrombotic syndrome with metabolic syndrome and inflammation - a systematic review

**DOI:** 10.3389/fimmu.2025.1519534

**Published:** 2025-03-28

**Authors:** Sara Alturky, Yusuf Ashfaq, Ajit Elhance, Megan Barney, Ishaq Wadiwala, Anna K. Hunter, Khanh P. Nguyen

**Affiliations:** ^1^ School of Medicine, Oregon Health & Science University, Portland, OR, United States; ^2^ Department of Surgery, Division of Vascular and Endovascular Surgery, Oregon Health & Science University, Portland, OR, United States; ^3^ Department of Pediatrics, Division of Gastroenterology, Oregon Health & Science University, Portland, OR, United States; ^4^ Division of Vascular Surgery, Research & Development, Portland Veterans Affairs (VA) Health Care System, Portland, OR, United States

**Keywords:** post-thrombotic syndrome, metabolic syndrome, deep vein thrombosis, venous thromboembolism, inflammation, vascular, obesity

## Abstract

**Introduction:**

Post-thrombotic syndrome (PTS) is a chronic complication of deep vein thrombosis (DVT). Given its impact on vascular health, understanding risk factors for the development of PTS, as well as conditions such as metabolic syndrome that may contribute to vascular inflammation, is crucial. Metabolic syndrome is a constellation of factors that increase cardiovascular disease risk, insulin resistance, diabetes mellitus (DM), and cerebrovascular disease. Despite the established connection between metabolic syndrome and venous thromboembolism (VTE), the association between metabolic syndrome and PTS has yet to be explored.

**Methods:**

A literature search identified studies regarding PTS and metabolic syndrome and the individual components of metabolic syndrome. A specialist performed the search, and studies were identified through PubMed, Ovid Medline, and Cochrane in accordance with PRISMA guidelines. Search terms included “post-thrombotic syndrome” and “metabolic syndrome” as well as “obesity,” “hyperglycemia,” “hypertension,” “dyslipidemia,” and “insulin resistance.” Two people independently screened articles and consolidated differences. Abstract-only studies, review articles, case studies, and conference abstracts were excluded. Case reports, literature reviews, and studies not discussing PTS were excluded. Prospective cohort, retrospective cohort, and case-control studies were included. All English-based studies that met inclusion criteria published before January 3rd, 2024, were included.

**Results:**

281 articles were initially identified. After abstract and title screening, 16 articles underwent full-text review. Of the 16 articles that underwent review, nine were included in the final analysis. Among the selected articles, eight out of nine mentioned obesity as a risk factor for developing PTS, making it the most common component mentioned. Hypertension, diabetes mellitus, hyperlipidemia, and low high-density lipoprotein (HDL) followed in prevalence. There was no noted difference between inflammatory markers in patients with and without PTS.

**Conclusion:**

Metabolic syndrome and its components, individually and in association with PTS, are not commonly examined. Eight articles examined the association of obesity with the development of PTS. This review identified a strong association between obesity, particularly abdominal or visceral obesity, and the development of PTS. While the association between PTS and VTE is established, further research is needed to identify the role of metabolic syndrome in the development of PTS.

## Introduction

Post-thrombotic syndrome (PTS), a long-term complication of deep vein thrombosis (DVT), is marked by chronic symptoms such as persistent leg pain, swelling, venous ulcers, and skin changes. These symptoms, which worsen with activity, can significantly diminish patients’ quality of life and impose a considerable burden on healthcare systems. It has been observed that 20-50% of individuals with DVT develop PTS, a condition that arises from residual venous obstruction and valvular reflux (1). The diagnosis of PTS is primarily clinical, based on a patient’s history of DVT and the presence of characteristic signs and symptoms. Validated tools, such as the Villalta score, evaluate symptom severity and clinical signs to stratify PTS into mild, moderate, or severe categories (2). The Villalta score is a widely recognized disease scale used for diagnosing and grading the severity of PTS (2). It evaluates five symptoms (pain, heaviness, cramps, paresthesia, and pruritus) and six clinical signs (pretibial edema, hyperpigmentation, venous ectasia, skin induration, and redness), scoring each from 0 (absent) to 3 (severe). A total score of ≥5 confirms PTS, with scores of 5–9 indicating mild PTS, 10–14 moderate PTS, and ≥15 or the presence of a venous ulcer denoting severe PTS. This standardized system is valued for its ability to monitor disease progression and assess treatment outcomes (2). Risk factors for PTS can be categorized into three groups: those present at the time of DVT diagnosis (including increased age, elevated body mass index (BMI), pre-existing primary venous insufficiency, history of ipsilateral DVT, and proximal DVT location), those related to the initial treatment (notably inadequate anticoagulation), and those identified during follow-up (such as recurrent DVT, residual thrombosis on imaging, and persistent elevation of D-dimer levels). It is unclear what factors are most critical for mitigating the risk of PTS. For those already affected, treatment focuses on symptomatic management through compression therapy, promoting healthy lifestyle choices, and, if necessary, surgical intervention (1).

Metabolic syndrome is a constellation of several disorders that may lead to an increased risk of developing cardiovascular disease, insulin resistance, diabetes mellitus (DM), and cerebrovascular disease (3). Metabolic syndrome is diagnosed if the patient has any three or more of the following conditions: waist circumference: >40 inches (males) & 35 inches (females), hypertension, >130/85 mmHg, or requiring drug treatment for elevated blood pressure, hyperglycemia: elevated fasting glucose >100 mg/dL or requiring drug treatment for elevated blood glucose, hypertriglyceridemia: elevated triglycerides >150 mg/dL or requiring drug treatment for elevated triglycerides, low high-density lipoprotein (HDL): <40 mg/dL (men) <50 mg/dL (women) or requiring drug treatment for low HDL cholesterol (4).

The etiology of metabolic syndrome is complex, involving genetic predisposition, corpulence with increased waist circumference, excess body mass, and a sedentary lifestyle. The root cause is the accumulation of excess adipose tissue, leading to tissue dysfunction and insulin resistance. The increased adipose tissue releases proinflammatory cytokines, including resistin, leptin, adiponectin, plasminogen activator inhibitor, and tumor necrosis factor, negatively affecting and modifying insulin sensitivity. Insulin resistance may be exacerbated by anomalies in the insulin receptor, diminished insulin production, and disruptions in the signaling cascade, impeding the body’s ability to utilize insulin efficiently. These impairments hinder the body’s ability to effectively use insulin, leading to a range of metabolic disturbances, notably metabolic syndrome, which can manifest as vascular injury and autonomic dysregulation (5, 6).

In the US, the prevalence of metabolic syndrome was estimated to be 93 million people in 2018 and rising (7). This multifaceted health condition increases the risk of many other disorders, particularly cardiovascular disease and type 2 DM (8). Moreover, metabolic syndrome has also been linked to VTE, which consists of DVT and pulmonary embolism (PE) (7). An extensive body of literature describes the relationship between metabolic syndrome and VTE, highlighting underlying causes of metabolic syndrome that can help mitigate the risk of this life-threatening condition.

Despite the purported connection between metabolic syndrome and VTE, little is known about the relation between metabolic syndrome and PTS (9). The pro-inflammatory environment that results from metabolic syndrome increases the lifetime risk of thrombosis (10). Given the health burden associated with PTS, it is essential to better understand their complex relationship and to identify potential risk factors that may contribute to the development of PTS. This systematic review seeks to explore and identify risk factors and underlying inflammatory mechanisms in patients with metabolic syndrome that may lead to the development of PTS.

## Methods

A comprehensive literature search was performed to identify all studies that discussed the development of PTS and metabolic syndrome or the individual components of metabolic syndrome in accordance with PRISMA guidelines. The electronic search was performed by a specialist, and studies were identified through Pubmed, Ovid Medline, and Cochrane.

### Inclusion and exclusion criteria

Only clinical articles specifically discussing PTS, metabolic syndrome, or its components in humans were included. Search terms included “post-thrombotic syndrome” and “metabolic syndrome” as well as “obesity,” “hyperglycemia,” “hypertension,” “dyslipidemia,” and “insulin resistance.” Articles discussing only chronic venous insufficiency and PTS in pediatric populations were excluded. Abstract-only studies, review articles, case studies, and conference abstracts were excluded. Case reports, literature reviews, and studies that did not directly discuss PTS were excluded. Prospective cohort, retrospective cohort, and case-control studies were included. All English-based studies that met the inclusion criteria published before January 3rd, 2024, were included.

### Screening and extraction

Two reviewers independently screened articles by abstracts and titles and collaborated to consolidate their differences. Disagreements were resolved by discussion of the articles with a third reviewer. For potential articles, the full text was retrieved and reviewed independently. Once the included studies were finalized, data was collected regarding study characteristics, patient demographics, comorbidities, and clinical outcomes.

## Results

**Figure 1:**
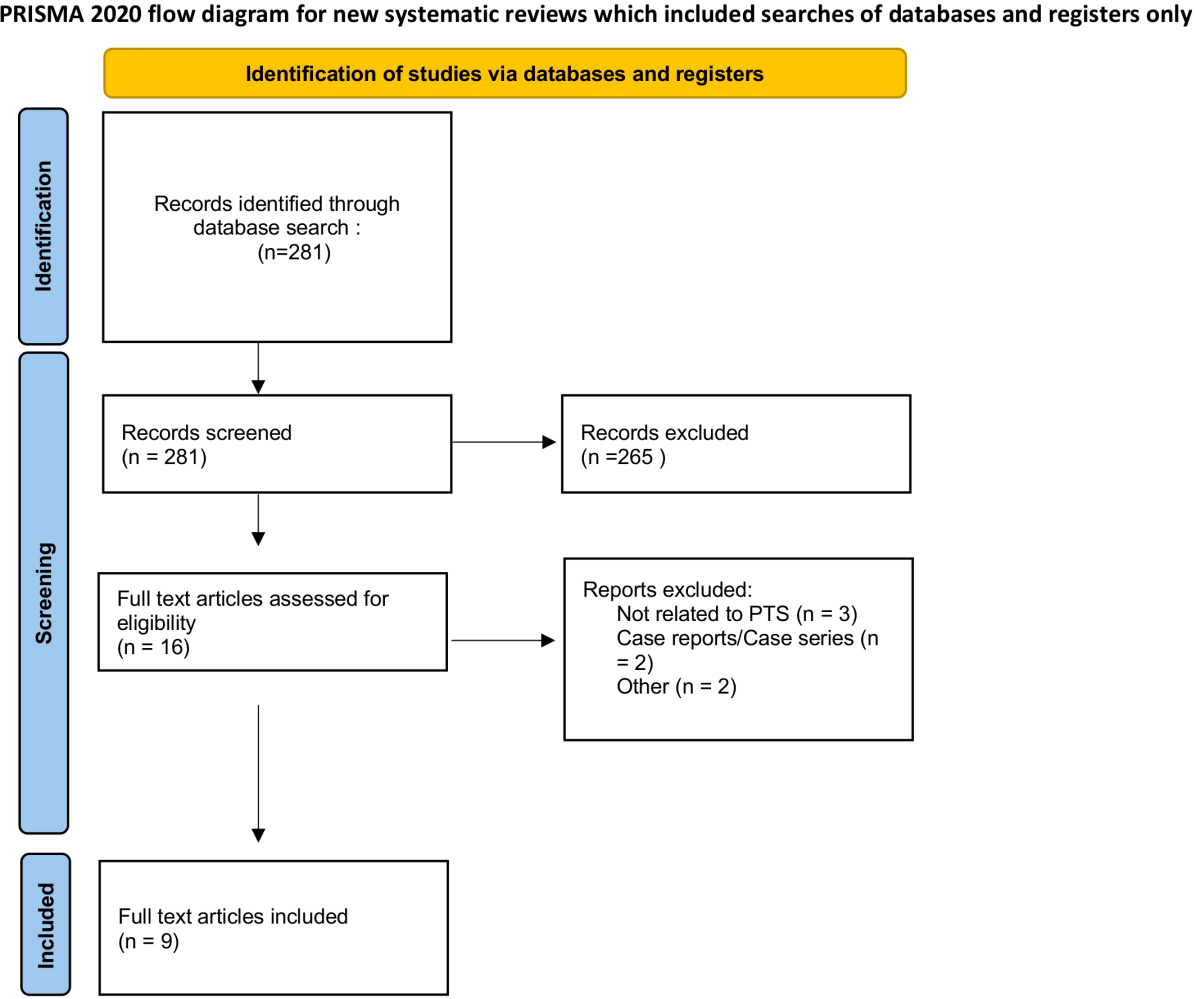
PRISMA flow diagram depicting the process of identifying, screening, and including studies in this systematic review.

There were 281 articles initially identified through our database search. After abstract and title screening, 16 articles underwent full-text review. Of the 16 articles that underwent full-text review, nine were included in the final analysis (**Figure 1**). The majority of the studies included were retrospective (n=5), followed by prospective cohort studies (n=3), and case-control studies (n=1). The characteristics of these papers are presented in **Table 1**. Of the nine studies included, three were multi-institutional. Across the studies included in this review, PTS was predominantly diagnosed using the Villalta score (**Table 2**). In addition to the Villalta score, some studies supplemented clinical assessment with imaging to detect residual thrombosis or venous obstruction. The Villalta score was used for diagnosis in 7 of the 9 included studies (12–18). Clinical examination was utilized to diagnose PTS in 6 of the 9 studies included (12–17). Ultrasonography was used in 4 studies (13–15, 19) and patient questionaries collecting data on symptoms and signs of PTS were utilized in 2 studies (13, 16). Color duplex sonography was utilized in three studies to objectively confirm DVT (12, 16, 19).

**Table 1:** Characteristics of studies included in the systematic review of the association of post-thrombotic syndrome with metabolic syndrome and inflammation.

First Author	PMID	Year	Study Design	Number of Patients	Males	Average Age	Average BMI	Obesity,n	PTS, n	Average Follow up length, months	Metabolic Syndrome Factors Mentioned	Key Findings
Mrozinska, Sandra	29720688	2018	Single institution Prospective Cohort	309	150	46	26.1	63	83	24	Obesity Diabetes Hypertension Hypertriglyceridemia Low HDL Inflammatory Markers	• Obese patients had higher leptin and lower adiponectin levels, which were independently associated with an increased risk of PTS regardless of obesity status. • While inflammation was implicated in PTS development, CRP levels and other inflammatory markers did not differ significantly between PTS and non-PTS groups. • Higher triglyceride levels were noted in patients with PTS, although the difference was modest.
El-Menyar, Ayman	28877605	2017	Single institution Retrospective Cohort	662	325	50.3	31	325	328	12	Obesity Diabetes Hypertension Hyperlipidemia	• Obesity was significantly associated with higher rates of recurrent DVT • PTS was observed in approximately 50% of patients, with similar rates across normal weight, overweight, and obese categories. This suggests that while obesity impacts other thrombotic risks, the likelihood of developing PTS itself may not vary significantly with weight. • Despite higher comorbidities, obese patients experienced lower mortality rates compared to non-obese individuals highlighting the “obesity paradox” in venous thromboembolism outcomes.
Ende-Verhaar, Yvonne	30870656	2019	Multi-institution Retrospective Cohort	1657	785	49	27	340	361	96	Obesity Inflammatory Markers	• The cumulative incidence of PTS was 22% at 1 year and an additional 7% between years 1 and 8 after a first DVT. • Obesity remained a consistent and significant risk factor for PTS over both short- and long-term follow-up. • Women were at higher risk of PTS than men, while older age (>60 years) appeared protective, with a lower risk compared to younger patients
Galanaud, J P	23279046	2013	Multi-institution Retrospective Cohort	328	187	52.7	not reported	131	89	7	Obesity Elevated Fasting Glucose*	• Among the 328 patients studied, 27.1% developed PTS within 5–7 months after their first DVT, with the majority having mild PTS. • Obesity was the strongest independent predictor of PTS
Asim, Mohammad	28527240	2017	Single institution Retrospective	662	325	50	not reported	257	328	12	Obesity Diabetes Hypertension Hyperlipidemia	• Obesity, dyslipidemia, diabetes, and abnormal coagulation were significant risk factors for recurrent DVT. • PTS was significantly more common in RDVT cases (58.2%) compared to single DVT cases (47.1%, p=0.01) • Advanced age, obesity, diabetes, and dyslipidemia were frequent comorbidities among RDVT patients.
Tick, L W	18983518	2008	Multi-institution Case-control	1668	788	48	27	1045	364	12	Obesity	• The 1-year cumulative incidence of PTS was 25%, with 7% of patients experiencing severe PTS • Obesity emerged as one of the strongest independent predictors of PTS, with a significantly higher cumulative incidence in obese patients. • Women, patients with pre-existing varicose veins, and those with proximal DVT in the femoral or iliac vein were at higher risk of PTS, while older patients (>60 years) had a lower risk compared to younger patients (<30 years).
Spiezia, Luca	34989625	2022	Single institute Prospective Cohort	769	353	59.8	27.2	40	152	36	Obesity	• Among the 769 patients with proximal DVT, 19.8% developed PTS within three years, with 3.9% classified as severe. • Obese patients had a significantly higher risk of PTS. • Age, gender, malignancy, unprovoked DVT, and thrombophilia were not significantly associated with PTS development in this cohort.
Rattazzi, M	26033396	2015	Single institution Retrospective Cohort	120	69	59	27.4	33	49	24	Obesity Diabetes Hypertension Hyperlipidemia HDL Inflammatory Markers	• The prevalence of metabolic syndrome was similar between patients with and without PTS • Waist circumference, a marker of visceral fat, was the only MetS component independently associated with PTS severity. • High-sensitivity CRP (hs-CRP) levels were not significantly different between groups, suggesting that visceral adiposity may contribute to PTS through mechanisms other than systemic inflammation.
Ageno, Walter	12574811	2003	Single institute Prospective Cohort Study	83	48	61.6	28.4	63	20	12	BMI**	• Patients who developed PTS had a significantly higher mean BMI (29.6 kg/m²) compared to those who did not (27.2 kg/m², p = 0.022). • A BMI >28 kg/m² was identified as an independent predictor of PTS.

**Table 1** presents key characteristics of studies included in this systematic review including the first author, PubMed identification number (PMID), year of publication, study design, number of patients included in the study, study demographics, and key findings.

*Elevated fasting glucose is listed as a component instead of diabetes because Galanaud et al. identified this marker specifically in relation to metabolic syndrome without explicitly diagnosing or categorizing patients as diabetic.

**BMI is listed instead of obesity because Ageno et al. specifically reported BMI as a continuous variable and identified a BMI >28 kg/m² as an independent predictor of PTS, rather than categorizing patients explicitly as obese or non-obese.

**Table 2:** Villalta scoring scale.

Category	Criteria	Grading Scale (Points)
Clinician Assessment	Pre-tibial Edema	Absent (0), Mild (1), Moderate (2), Severe (3)
	Skin Induration	Absent (0), Mild (1), Moderate (2), Severe (3)
	Hyperpigmentation	Absent (0), Mild (1), Moderate (2), Severe (3)
	Redness	Absent (0), Mild (1), Moderate (2), Severe (3)
	Venous Ectasia	Absent (0), Mild (1), Moderate (2), Severe (3)
	Pain on calf compression	Absent (0), Mild (1), Moderate (2), Severe (3)
Patient Symptoms	Pain	Absent (0), Mild (1), Moderate (2), Severe (3)
	Cramps	Absent (0), Mild (1), Moderate (2), Severe (3)
	Leg Heaviness	Absent (0), Mild (1), Moderate (2), Severe (3)
	Paresthesia	Absent (0), Mild (1), Moderate (2), Severe (3)
	Pruritis	Absent (0), Mild (1), Moderate (2), Severe (3)
	Venous ulcer present	Absent (0), Present (15)

**Table 2** presents the Villalta scoring scale which is divided into both clinician assessment and patient symptoms. These factors are then scored from 0-3 (absent to severe) with classifications of mild if the score is 5-9, moderate if 10-14, and severe if ≥15 or the presence of a venous ulcer (11).

Sample sizes in the included studies ranged from 83 to 1,668 patients. A total of 6258 patients were included in the studies, 3030 (48%) of whom were male (**Table 3**). Ages reported ranged from 18 – 97, and the mean age of all patients was 52.93 ± 5.40 years. The mean BMI of all patients was 29.28 ± 2.11 kg/m^2^. Notably, all nine articles reference obesity as a risk factor for developing PTS, and obesity was noted in 2297 patients (37%). The average length of follow-up for these patients was 26.38 ± 27.81 months (range 7- 96 months).

**Table 3:** Pooled patient characteristics from literature review.

	n (%)	Mean ± SD	Range
Gender	6258 (100)		
Male	3030 (48.0)		
Female	3228 (52.0)		
Age (years)	6258 (100)	52.93 ± 5.40	18-97
BMI (kg/m^2^)	4959 (100)	29.28 ± 2.11	13-66
Metabolic Syndrome Components			
Obesity	2297 (36.7)		
Hypertension	629 (10)		
Diabetes	400 (6.4)		
Hypertriglyceridemia	268 (4.3)		
Low HDL	13 (0.2)		
Metabolic Syndrome as a whole	20 (3.2)		
PTS*	1774 (100)		
Obesity	625 (35.2)		
Hypertension	50 (2.8)		
Diabetes	8 (0.5)		
Metabolic Syndrome as a whole	20 (1.1)		
Average follow-up (months)		26.38 ± 27.81	7-96

**Table 3** presents the population characteristics of the studies included in this review. This table lists the percent of male and female patients, average and standard deviation for age, average and standard deviation for BMI, number of patients with obesity, number of patients with hypertension, number of patients with hypertriglyceridemia, number of patients diagnosed with PTS, and number of patients diagnosed with metabolic syndrome. This table also presents the components of metabolic syndrome found in patients with PTS. No studies reported how many patients with PTS had hyperlipidemia and low HDL.

*Numbers presented for each component are derived from the studies that reported on them, however not all studies provided data for every component.

Of the components of metabolic syndrome, obesity was the most common component noted in patients. This was followed by hypertension (n=629, 10%), DM (n=400, 6%), hypertriglyceridemia (n=268, 4%), and then low HDL (n=13, 0.2%). 1,774 patients (28%) were noted to have PTS.

Of the 1774 patients with PTS, gender was reported for 645, of which 273 (42%) were men (**Table 4**). The average age for patients with PTS was 54.5 ± 9.1 years, and the average BMI was 28.45 ± 0.95 kg/m^2^, compared to an average age of 52.8 ± 8.2 years and BMI of 26.58 ± 0.75 kg/m^2^ for patients without PTS. Obesity was reported in 625 patients (35.2%) with PTS, as opposed to 1450 (34.7%) without PTS. A total of 747 patients were diagnosed with PTS using the Villalta scale. Among these, 135 patients had severe PTS, defined by a Villalta score of ≥ 15, while 662 were classified as having mild to moderate PTS. Among studies utilizing the Villalta scale, Rattazzi et al. (15) reported that patients with PTS had an average Villalta score of 7.5 ± 2.7, compared to 2.3 ± 1.1 in patients without PTS, highlighting the significant difference in clinical severity between these groups.

**Table 4:** Comparing those with and without PTS.

	PTS (n = 1774)	No PTS (n = 4484)
Sex (n=645)		
Male (%)	273 (20.1)	1084 (79.9)
Female (%)	372 (24.8)	1126 (75.2)
Mean Age (years)	54.5 ± 9.1	52.8 ± 8.2
Mean BMI (kg/m²)	28.45 ± 0.95	26.58 ± 0.75
Metabolic Syndrome (n)	20	26
Components of Metabolic Syndrome		
Obesity, n (%)	625 (30)	1450 (70)
Hypertension, n (%)	50 (35)	93 (65)
Diabetes, n (%)	8 (32)	17 (68)
Mean Triglyceride (mg/dL)	105.3	106.4
Mean HDL (mg/dL)	56.4	57.5
Mean CRP (mg/L)	1.55	2.45
Mean D Dimer (ng mL^−1^)	287	277

**Table 4** presents demographic and clinical characteristics of patients with and without PTS, including sex distribution, mean age, BMI, and components of metabolic syndrome.

Rattazzi et al. (15) presented the only study that directly assessed the relationship between patients diagnosed with metabolic syndrome and PTS. 120 patients were evaluated, and of the 49 patients reported to have PTS, only 20 (41%) also had metabolic syndrome.

Inflammatory markers were evaluated in 4 of the 9 studies included (44%) (12–15). These comprised adiponectin, leptin, resistin, plasminogen activator inhibitor- 1, D-dimer, fibrinogen, and C-reactive protein (CRP) levels. Of patients with reported CRP, the average CRP for patients with PTS was 1.55 mg/L, compared to 2.45 mg/L for patients without. D-dimer was only reported in one study, and the average reported for patients with PTS was 287 ng mL^−1^, compared to 277 ng mL^−1^ for those without PTS (12). This difference was insignificant, and the reference range was (226-337) ng mL^−1^.

## Discussion

PTS is a chronic condition that often develops following DVT. While the exact causes are not fully understood, the existing literature suggests inflammation plays a key role in its onset (20). A history of DVT contributes to the development of PTS, as the thrombus can damage valves, leading to venous obstruction and hypertension, which presents as pain, swelling, discoloration, and ulceration (21). The initial inflammation is responsible for the thrombus resolution by clot degradation, which simultaneously promotes collateral damage to the venous tissue and valves. This chronic inflammation may have a role in PTS (20). Inflammatory adipokines, such as leptin and adiponectin, play a significant role in this process. Elevated leptin levels promote platelet aggregation and enhance plasminogen activator inhibitor-1 (PAI-1), impairing fibrinolysis, while lower adiponectin levels mitigate these effects by reducing oxidative stress and promoting nitric oxide production. These adipokines have been shown to predict PTS independently of obesity, underscoring their unique contribution to venous pathophysiology (12). Individuals with chronic inflammation are at a higher risk of experiencing recurrent DVT. This hypercoagulable state may be attributed to the complex interactions between inflammatory processes and the coagulation system triggered by ongoing low-grade inflammation (22). Residual venous obstruction and underlying chronic inflammation are common findings in patients with PTS.

**Figure 2:**
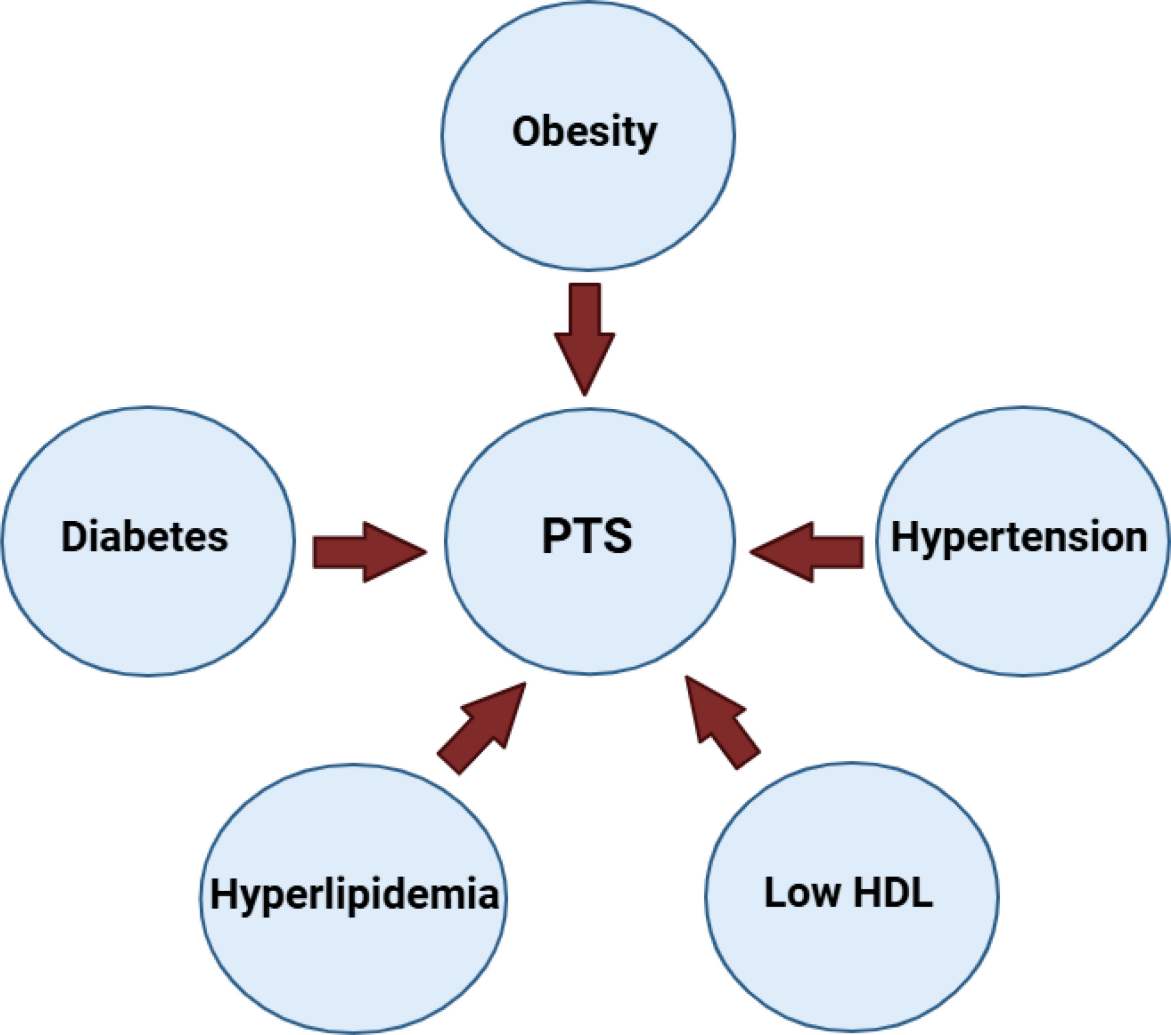
Diagram depicting potential risk factors for post-thrombotic syndrome (PTS).

Metabolic syndrome is comprised of a combination of three or more of the following: obesity, hypertension, hyperglycemia, hypertriglyceridemia, and low HDL. Metabolic syndrome increases the risk of developing conditions such as cardiovascular disease, DM, and cerebrovascular disease. The underlying mechanism of metabolic syndrome involves a combination of genetics and lifestyle factors, with inflammation resulting from excess adipose tissue playing a crucial role. The components of metabolic syndrome and related inflammation can influence the development of PTS (**Figure 2**). Our discussion below breaks down the current literature regarding each component of metabolic syndrome and its association with the development of PTS.

### Obesity

In our review, eight of the nine included studies discussed the relationship between obesity and the development of PTS (12–19). Ageno et al. (18) was the only study that did not directly mention obesity as a contributor—but rather BMI as a driving factor in the development of PTS. In Rattazzi et al.’s (15) assessment of metabolic syndrome and its components’ relationship with PTS, they discovered that obesity, measured by visceral adiposity, significantly increases the likelihood of developing PTS. Waist circumference, a marker of visceral fat, has been strongly correlated with PTS severity as measured by the Villalta score (15). This metric serves as both an indicator of obesity and a contributor to inflammatory pathways that exacerbate venous hypertension and remodeling, directly driving PTS progression (15). This finding reinforces obesity as a driving factor for PTS, even in the absence of other components of metabolic syndrome. Interestingly, one of the studies by El-Maynar et al. (23) found that although obesity contributes to the development of DVT and the pro-inflammatory state, the incidence of PTS was similar between the obese and non-obese groups. This suggests that while obesity increases the risk for DVT, it does not necessarily lead to a higher risk of developing PTS. The remaining six studies, however, further demonstrated that obesity is associated with an increased risk of PTS (12–14, 16, 17, 19). The relationship between obesity, inflammation, and coagulation emphasizes the prothrombotic state resulting from increased adipose tissue. Given this, it is understandable that obesity acts as a key risk factor for the development of thrombotic sequala, such as VTE and DVT, and the progression to PTS and appears to drive a localized inflammatory response (15).

Abdominal or visceral obesity, a marker of metabolic syndrome is reported to lead to stasis of blood, which is a precursor to DVT (24). The risk of developing recurrent DVT rises by 25% with every 10-point increase in BMI (25). This was reinforced in our review by El-Meynar et al. (23), whose study demonstrated that individuals with obesity are more prone to thrombotic events. Similarly, another study demonstrated a 2-fold increase in recurrent DVT with a BMI of over 30 compared to normal BMI (26). Obesity affects the coagulation cascade by decreasing fibrin degradation and increasing clotting factors (27). Additional findings further support this by demonstrating elevated levels of d-dimer, fibrinogen, coagulation factor VIII, and coagulation factor IX significantly correlated to BMI (28). These coagulation changes are likely mediated by the metabolic activity of adipose tissue, which secretes bioactive molecules that influence both systemic and localized hemostatic balance. This can be explained by the release of adipokines from excess fatty tissue that shifts the pendulum from an antithrombotic to a prothrombotic state (29). Obesity can also be considered a chronic low-grade inflammatory condition where the proinflammatory factors secreted by adipose tissue increase. Weight loss diminishes this proinflammatory state conferred by excess fatty tissue and clotting factors (30).

### Hypertension

The role of hypertension in PTS remains unclear. El-Meynar et al. (23) noted that hypertension is more prevalent in obese patients and is one of the leading cardiovascular risk factors that contribute to DVT. Similarly, Rattazzi et al. (15) also addressed the relation between hypertension and its contribution to overall cardiovascular and thrombotic risk. However, in both aforementioned studies, it was noted that there is not an observed association between high blood pressure and the development of PTS, as rates of hypertension were equivalent between patients with and without PTS. Asim et al. (19) further concluded that while hypertension was prevalent in DVT patients (37%) and in those with recurrent DVT (38.4%), it was not a statistically significant independent risk factor for DVT recurrence.

Hypertension is associated with a higher risk of VTE (31, 32). Hypertension may contribute to hemostasis, endothelium dysfunction, and vessel inflammation, leading to an increased risk of thrombosis and ultimately a pro-inflammatory state. Inflammation subsequently leads to the stiffening of blood vessels and vascular remodeling, and these processes play a critical role in the development of PTS (15, 33). Pro-inflammatory cytokines, including interleukin-6 (IL-6) and tumor necrosis factor-alpha (TNF-α), promote oxidative stress, leukocyte adhesion, and the recruitment of macrophages, which release matrix metalloproteinases (33). These enzymes degrade the extracellular matrix, contributing to structural alterations in the vascular wall. Additionally, endothelial cells, under inflammatory stress, increase the expression of adhesion molecules like vascular cell adhesion molecule 1 (VCAM-1) and intercellular adhesion molecule-1 (ICAM-1), further amplifying leukocyte infiltration and local inflammatory responses. This cascade perpetuates vascular remodeling through smooth muscle cell proliferation, collagen deposition, and impaired nitric oxide bioavailability, all of which contribute to vessel stiffening and venous dysfunction (33). In the context of metabolic syndrome, hypertension frequently coexists with other risk factors, such as obesity and hyperlipidemia, yet its direct role in PTS remains less clear, with systemic effects like inflammation and endothelial dysfunction taking precedence (23). Hypertension has also been linked to an increased risk of recurrent VTE, which indirectly elevates the risk of developing PTS by compounding venous damage and obstruction (16). Our results suggest that while hypertension and obesity often coexist in patients resulting in an increased risk of DVT, the role of hypertension in PTS may hinge on additional factors.

### Hyperglycemia

Type 2 DM is a chronic disease of hyperglycemia and is a known risk factor for VTE, which includes PE and DVT (31, 34, 35). Three studies included in our review discussed the impact of DM. El-Meynar et al. (23) highlighted how DM, along with other metabolic conditions, contribute to the development of a prothrombotic state, thereby increasing the risk for DVT and PTS. While no direct association between the conditions was made, Asim et al. (19) identified DM as a comorbid risk factor for recurrent DVTs. Conversely, Rattazzi et al. (15) found no association between DM and the development of PTS.

Acute hyperglycemia is associated with a poorer prognosis in thrombosis (36). In DM, hyperglycemia may lead to a hypercoagulable state, and both acute and chronic hyperglycemia increase the risk for VTE (36). Galanaud et al. (14) shows that elevated fasting glucose levels, even without a diagnosis of diabetes, has been linked to heightened inflammatory and thrombotic states. This is measured by elevated levels of inflammatory markers such as CRP and IL-6, as well as thrombotic markers like D-dimer and fibrinogen. This suggests that glucose dysregulation may exacerbate vascular complications associated with PTS. In chronic hyperglycemia, many physiologic changes result in a prothrombotic state; this includes a decrease in activated partial thromboplastin time, prothrombin time, protein C, antithrombin, and fibrinolysis, and a paradoxical increase in clotting factors II, VII, VIII, fibrinogen, and tissue factor (37–41). Glucose metabolism dysfunction may also affect residual venous obstruction, contributing to long-term PTS progression (14). Correction of hyperglycemia, irrespective of treatment modality, reduced thrombotic events in patients with DM (42). Many hypotheses exist that try to explain the relationship between hyperglycemia and hypercoagulability (43). Elevated glucose and insulin increase levels of plasminogen activator inhibitor activity, which reduces tissue plasminogen activator and subsequent fibrinolysis (44). Exposure of advanced glycation end products to endothelial cells causes activation of the coagulation cascade, and procoagulant activity is seen (45). In type I DM, when insulin is absent, tissue factor and plasminogen activator inhibitor levels surge. These elevations are accompanied by malondialdehyde and protein carbonyl groups, which are surrogates for oxidative stress (46). These changes may lead to an overall pro-inflammatory and pro-thrombotic state but have not been shown to increase an individual’s risk for the development of PTS in the existing literature.

### Hyperlipidemia

Four studies included in our review assessed lipid profiles. Asim et al. (19) observed that hyperlipidemia and dyslipidemia were once again noted to be more common in patients with recurrent DVT, with 34.9% of patients with recurrent DVT having dyslipidemia compared to 25.8% in single DVT cases. El-Meynar et al. (23) found that dyslipidemia and hypertriglyceridemia were more commonly observed in obese patients, suggesting that the disruption in fat metabolism was contributing to the prothrombic state. Similarly, Mrozinska et al. (12) noted that patients who developed PTS had higher triglyceride levels compared to those without, highlighting the potential role triglycerides may contribute to a prothrombotic environment. This was however contradicted in the study by Rattazzi et al. (15) who found no association between lipid levels and the development of PTS. In addition, no statistically significant differences were found between groups treated with a statin and those without (15).

Hypertriglyceridemia is recognized for elevating blood viscosity, which can lead to a procoagulant state (47). This effect is primarily linked to venous thrombosis. The pro-thrombotic effects of elevated triglycerides involve several mechanisms: enhanced platelet aggregation, reduced activity of antithrombin III and interactions with coagulation factors, a rise in proinflammatory markers, and endothelial dysfunction (48). In addition to increasing blood viscosity, triglycerides increase plasminogen activator inhibitor and factor VII (49). Triglyceride-rich lipoproteins (TRL) in the blood are considered risk factors and contribute to atherosclerosis and inflammation (49, 50). TRLs interact with coagulation factors, promote endothelial dysfunction, and contribute to chronic low-grade inflammation. This effect is largely mediated by increased expression of vascular adhesion molecules and tissue factors, as observed by multiple studies (15, 18, 23). These particles accumulate in the vessels and are not cleared as frequently (45). In the context of atherosclerosis, foam cells—lipid-laden macrophages—accumulate within arterial walls, contributing to plaque formation. These foam cells upregulate metalloproteinases, enzymes that degrade extracellular matrix components, leading to the erosion of the fibrous cap covering the plaque (48). This degradation weakens the cap, increasing the risk of plaque rupture and subsequent thrombus formation (2). Additionally, TRLs achieve this prothrombotic state by directly increasing intercellular adhesion molecule-1, vascular cell adhesion molecule-1, and tissue factor in endothelium lining (51). These molecules facilitate leukocyte adhesion and transmigration, further promoting inflammation and thrombogenesis (45, 48). Elevated blood levels of triglycerides result in aberrant platelet overactivity (52). Increases in triglycerides above 200 mg/dL have been associated with a decrease in antithrombin activity and increased platelet aggregation (52).

### High-density lipoprotein

Rattazzi et al. (13) was the only study that assessed HDL levels and their impact on the development of PTS and found no significant association between low HDL and the development of PTS, however, it suggests that the atherogenic lipid profile, defined by low HDL and high triglycerides, could play a potential role in the development of PTS.

HDL is a lipoprotein, and increased levels of HDL confer protection against cardiovascular disease (53). Elevated HDL levels would reduce the risk of developing DVT (54). HDL is known to have antithrombotic effects on blood vessels (55, 56). Additionally, patients with low HDL levels were more likely to develop DVT (31). This antithrombotic effect may be explained by reducing platelet aggregation and activation of the coagulation cascade. HDL also increases the production of endothelial prostacyclin and nitric oxide, which inhibits platelet aggregation, deactivates clotting factors, and reduces tissue factor production (57). Ultimately, thrombin is decreased, which reduces fibrin conversion from fibrinogen, reducing platelet aggregation (58).

### Inflammatory and metabolic associations

Inflammation plays a role in many components of metabolic syndrome, and this was discussed in several studies. Rattazzi et al. (15) compared the prevalence of metabolic syndrome in patients with and without diagnosed PTS. While no significant difference in the overall prevalence of metabolic syndrome was found between the two groups, a strong association between visceral adiposity and both the presence and severity of PTS was identified, suggesting that visceral fat, rather than metabolic syndrome, is more closely linked to PTS. This association was further emphasized by a linear correlation between the Villalta score, which measures PTS severity, and waist circumference, a marker of visceral obesity, underscoring the role of localized fat accumulation in driving inflammation and PTS development (15). This was the only article that assessed the two chronic conditions together (15).

This finding was further supported by Mrozinska et al. (12), who found that lower adiponectin, which has anti-thrombotic effects, and higher leptin, which promotes platelet aggregation, measured three months after DVT, despite obesity or inflammatory states, independently predicted the development of PTS. In the article, leptin was identified as a key pro-inflammatory and pro-thrombotic adipokine, promoting platelet aggregation and enhancing the production of PAI-1, which impairs fibrinolysis. Conversely, adiponectin was shown to protect against thrombosis by reducing platelet activation and mitigating oxidative stress through the promotion of endothelial nitric oxide production. This imbalance of adipokines suggests that inflammation and thrombosis in PTS are influenced by adipose tissue beyond general obesity metrics. This interplay between adipokines and inflammation highlights how obesity-driven chronic inflammatory states may exacerbate the risk of PTS, even in patients without overt metabolic syndrome. All four studies that discussed inflammatory markers concluded that inflammatory markers such as CRP and D-dimer do not predict the development of PTS (12–15).

Ende-Verhaar et al. (13) investigated the role of inflammation over an 8-year period in patients diagnosed with PTS and found that, although CRP is an inflammatory marker, it did not have a significant predictive value for PTS when compared to factors such as obesity and thrombus location. This study highlighted that obesity, particularly visceral obesity, and recurrent DVT were stronger drivers of PTS than chronic systemic inflammation alone. The findings suggest that inflammatory biomarkers like CRP may reflect generalized inflammation without capturing localized processes, such as endothelial dysfunction and venous remodeling, that are critical to PTS. Tick et al. (16) expanded on this concept, emphasizing that venous valvular incompetence and persistent venous obstruction create a localized inflammatory state that is significant in PTS progression. Obesity amplified these effects, increasing the 1-year cumulative incidence of PTS from 22% in normal-weight patients to 34% in obese individuals (16).

Metabolic syndrome is also linked to a hypofibrinolytic and procoagulant state. Clot lysis time is considerably increased in patients with metabolic syndrome compared to healthy controls (59). Metabolic syndrome may alter the production of adipokines due to excess adipose tissue, which leads to low-grade chronic inflammation responsible for endothelial dysfunction, vascular remodeling, and thrombosis (24, 25, 60). This aligns with the observations of Rattazzi et al. (15) and Mrozinska et al. (12), who identified visceral fat and adipokine dysregulation as pivotal factors in PTS pathogenesis. The pathophysiology of idiopathic VTE may involve all the components of metabolic syndrome, which may also serve as a link between atherosclerosis and VTE (31).

Inflammation is crucial in thrombus formation, mediated by inflammatory pathway activation and recruitment of platelets and leukocytes. Thrombosis and inflammation are distinct systems but have significant overlap (61). Under physiological circumstances, inflammation activates the coagulation system, a component of the body’s natural reaction to pathogens, ultimately suppressing their spread through blood. This process, known as immunothrombosis, involves leukocytes and platelets that activate the coagulation cascade (62). In PTS, this inflammatory response may persist or become dysregulated, leading to chronic endothelial activation, venous hypertension, and impaired fibrinolysis, which drive the progression of post-thrombotic changes. Galanaud et al. (14) underscored that residual venous obstruction, a hallmark of PTS, is exacerbated by chronic inflammation and inadequate anticoagulation, further worsening venous stasis and thrombus burden.

Inflammation activates thrombin, which in turn affects endothelial cells, smooth muscle cells, and platelets. Protease-activated receptors are also actuated by thrombin. This increases endothelium expression of vascular cell adhesion molecules, intracellular adhesion molecules, P-selectin, and E-selectin. These molecules facilitate leukocyte and platelet adhesion, amplifying local inflammation and thrombus stabilization, which may persist in PTS. Chemokine production by the vascular endothelium is also increased, including monocyte chemotactic protein-1, platelet-derived growth factor, and interleukins (60). Platelets also express CD40 ligands, which activate leukocytes that have the CD40 receptor (60). It was noted by Asim et al. (19) that recurrent DVT makes matters complex as there are increased inflammatory markers and abnormal coagulation profiles in patients with repeated thrombotic episodes which lead to higher rates of PTS. This interplay further highlights the significant overlap between vascular remodeling, inflammation, and thrombosis in the context of metabolic syndrome and PTS. Addressing the underlying inflammation could provide preventative therapeutic opportunities.

### Follow up periods

Long-term follow-up periods beyond one year provided valuable insights into the progression and complications of PTS. Of the studies included, eight of the nine had follow-up periods of one year or greater (12, 13, 15–19, 23). Ageno et al. (18) observed that the cumulative incidence of PTS increased progressively over time, reaching 30.3% by eight years, with symptoms such as leg heaviness, varicose veins, and venous ulcers becoming more pronounced in some patients. Interestingly, while some individuals improved in their PTS classification, others experienced worsening symptoms, with 13 patients in Ageno et al.’s (18) cohort progressing from moderate to severe PTS over eight years. Tick et al. (16) reported that severe PTS occurred in 7% of patients within one year and noted that women, obese individuals, and those with proximal DVT faced higher risks, reflecting the impact of these risk factors on disease severity. Spiezia et al. (17) found that obesity and iliofemoral DVT were significant predictors of PTS over a three-year follow-up, and patients with severe PTS often presented with persistent leg swelling, pain, and venous ulcers despite treatment. Rattazzi et al. (15) also emphasized the impact of excess adipose tissue on venous health through the link between visceral adiposity and persistent venous inflammation and obstruction, as observed in patients assessed more than two years after their initial DVT diagnosis. El-Menyar et al. (23) highlighted recurrent DVT as a significant complication associated with PTS, noting that while the link was strong initially, its impact diminished after adjusting for confounders, such as the duration of anticoagulation therapy. Overall, longer follow-up periods help in understanding the progression of disease as well as the impact of risk factors in patient outcomes.

### Potential risk factors

Several studies highlighted key risk factors for PTS, emphasizing the interaction between metabolic syndrome, vascular inflammation, and other clinical features (12–19, 23). Visceral adiposity, obesity, and elevated BMI were consistently identified as risk factors for PTS and associated with earlier onset of PTS. However, while weight gain post-DVT was less directly correlated with PTS development, it remains an important metabolic consideration due to its broader implications for vascular health (18). Reduced physical activity was also implicated, as it can hinder a muscle’s ability to efficiently pump blood, compounding venous insufficiency and increases the risk of PTS, although detailed assessments of activity levels were limited (13). Proximal thrombus location and recurrent DVT were identified as significant contributors, with both factors worsening venous damage and elevating the risk of PTS progression (16). Demographic factors, such as higher BMI and female sex, were linked to increased PTS risk, though the impact of sex differences diminished over longer follow-up periods (17). Inadequate anticoagulation is a significant risk factor in the development of PTS. Subtherapeutic anticoagulation or residual venous obstruction due to poor INR control, as highlighted by Galanaud et al. (14), significantly elevated the risk of developing PTS. This was in part due to patient adherence to prescribed anticoagulants. These risk factors remain areas for potential improvement in order to enhance patient outcomes.

### Protective factors

Protective factors for PTS were less commonly reported. Compression stockings were noted as a potential intervention in some studies, but their effectiveness in preventing PTS remains inconsistent. While studies like Tick et al. (16) reported widespread use, PTS still developed in 25% of cases within one year despite daily compliance, suggesting limited efficacy in some populations. Conversely, Ende-Verhaar et al. (13) noted potential benefits when compression stockings were used consistently over extended periods, but the results varied across follow-up durations. Both Ende-Verhaar et al. (13) and Tick et al. (16) identified older age (>60 years) as a protective factor, with older individuals demonstrating a significantly lower risk of developing PTS compared to younger patients (<30 years). As previously mentioned, effective anticoagulation remains a key protective risk factor for PTS (14). In addition, weight management remains a key preventative measure despite the “obesity paradox” demonstrated by El-Menyar et al. (23), where a lower mortality rate was suggested for patients with obesity despite their inherent increased risk for DVT and PTS. Lastly, symptom improvement over time, as noted by Ende-Verhaar et al. (13), highlights the potential for natural resolution in some cases.

### Medical treatment options

While there is limited direct evidence regarding the potential effects of ongoing treatments for PTS, there are a few studies that have explored this topic. Rattazzi et al. (15) emphasized that weight loss strategies could play a significant role in mitigating PTS severity by reducing visceral adiposity. Pharmacological treatments, such as statins and antihypertensive therapies, were also mentioned as potential confounders in PTS progression, but their specific impact on metabolic syndrome parameters was not definitively assessed (15). As mentioned previously, the dysregulation of adipokines is implicated in chronic venous disease and PTS. These may be a possible target for treatment through lifestyle or medication management which could benefit both conditions (12).

Beyond metabolic syndrome, the role of DVT treatment and anticoagulation therapy in managing inflammation is essential. Coagulation factors, such as thrombin and factor Xa, not only contribute to clot formation but also act as mediators of inflammation by activating protease-activated receptors (PARs) and promoting cytokine release (63). The use of factor Xa inhibitors, such as rivaroxaban and apixaban, has been shown to suppress inflammatory pathways by inhibiting PAR signaling, potentially reducing cytokine-driven endothelial damage (64). Similarly, warfarin, while primarily an anticoagulant, has demonstrated secondary effects on inflammatory markers like IL-6 and CRP (64). Low-molecular-weight heparins (LMWH) have been highlighted for their additional anti-inflammatory properties, which are thought to influence PTS prevention by reducing venous wall inflammation, improving endothelialization, and reducing fibrosis. Studies have suggested that LMWH may also lead to better rates of venous recanalization compared to traditional Vitamin K antagonists (VKAs) by acting on inflammatory markers involved in thrombosis pathways (65, 66). Lastly, compression therapy, as mentioned previously, is often recommended as a first-line option for managing PTS, despite studies showing varying efficacy (65, 66). Based on these findings it seems that anticoagulation therapy influences both thrombus formation and inflammatory pathways, providing a multifaceted benefit.

### Surgical treatment options

In addition to prevention and medical management, surgical treatment options following DVT have been explored and researched with the hopes of reducing the risk of PTS. Open thrombectomy and endovascular thrombolysis are two options aimed at restoring venous patency and reducing venous hypertension however their efficacy in preventing PTS remains uncertain (67). Open thrombectomy, while previously utilized, is no longer considered routine treatment for DVT and has been largely replaced by less invasive procedures such as catheter-directed therapies, which often provide comparable efficacy with reduced morbidity and surgical complications (68). Catheter-directed therapies were further evaluated in the Acute Venous Thrombosis: Thrombus Removal with Adjunctive Catheter-directed Thrombolysis (ATTRACT) trial, a large multicenter randomized study that investigated whether pharmacomechanical catheter-directed thrombolysis could prevent the development of PTS in patients diagnosed with acute proximal DVT. This trial however found no significant reduction in PTS rates among patients treated with catheter-directed thrombolysis compared to anticoagulation alone (67). While short-term improvements in pain and swelling in patients with DVT were observed, these benefits did not translate into long-term prevention of PTS, and an increased risk of bleeding and serious complications such as intracranial hemorrhage was noted (67). The Catheter-directed Venous Thrombolysis Trial (CaVenT), a prospective, multicenter, randomized controlled trial, also evaluated the use of catheter-directed thrombolysis in patients with proximal DVT to assess its potential in reducing PTS incidence (69). Interestingly, the CaVenT study determined that traditional anticoagulation with additional catheter-directed thrombolysis did result in a clinically significant reduction of PTS in patients however it similarly reported a heightened risk of bleeding complications (69).

Considering these findings, use of surgical intervention remains largely controversial and is not recommended solely for the prevention of PTS and alternative medical management, such as compression therapy and anticoagulation remain first line (67). Careful patient consideration and individualized decision-making is crucial when contemplating surgical management for patients with DVT.

### Future directions

To our knowledge, this is the first systematic review addressing the association between metabolic syndrome and its components and the development of PTS. Currently, there is limited information investigating this relationship, however data exists suggesting that the association between the two conditions are likely driven by changes in inflammatory and prothrombotic factors. As there are current gaps in knowledge, larger retrospective studies with longer follow-up periods would aid in our understanding of the relationship between metabolic syndrome components and PTS. In addition, sub-analysis of patient populations can help in stratifying associations by age, gender, and additional comorbidities. A meta-analysis may be considered in the future.

## Limitations

This systematic review was limited by the relatively small number of studies that have investigated the association between metabolic syndrome and its components, as well as PTS. Despite collecting and analyzing all available studies, the total number of studies was small. Additionally, while this review included 9 articles, only one article discussed metabolic syndrome as a whole and its association with PTS, while the remaining articles discussed the individual components. This limits our ability to draw definitive conclusions about the relationship between metabolic syndrome as a whole and PTS but instead focuses on the individual components that may be seen in patients with metabolic syndrome. There was also high heterogenicity in the studies included, limiting our ability to generalize findings. The assessment for PTS was performed at different follow-up times in all studies, which introduced variability and could impact the analyses. Finally, while included studies likely were referencing type 2 diabetes mellitus, it was not explicitly distinguished from Type 1, and this can be explored in future investigations.

## Conclusion

While only one existing study directly investigates metabolic syndrome and its association with PTS, several papers highlight how the individual components of metabolic syndrome increase inflammation and, thereby, the risk of thrombosis. The association between PTS and VTE is established, however, further research is needed to clarify the role of metabolic syndrome and its components in combination in the development of PTS. Nonetheless, this systematic review identified a strong association between obesity, particularly abdominal obesity, and the development of PTS.

## Data Availability

The original contributions presented in the study are included in the article/supplementary material. Further inquiries can be directed to the corresponding author.
